# Study of TiAlN PVD Coating on Stamping Dies Used in Tinplate Food Package Production

**DOI:** 10.3390/mi10030182

**Published:** 2019-03-12

**Authors:** Liliana Fernandes, Francisco J. G. Silva, Ricardo Alexandre

**Affiliations:** 1 ISEP—School of Engineering, Polytechnic of Porto, Rua Dr António Bernardino de Almeida, 431, 4200-072 Porto, Portugal; lpbfernandes@hotmail.com; 2 TEandM—Technology, Engineering and Materials, S.A., Parque Industrial do Taveiro, Lotes 41/42, 3045-508 Taveiro, Portugal; ricardo@teandm.pt

**Keywords:** tinplate, PVD coatings, molds and dies, stamping, wear behavior, friction, food packages

## Abstract

The food industry is increasingly demanding in terms of the quality and appearance of its product packages. The present study focuses on identifying the main wear mechanisms developed during the stamping process of these packages. During the stamping process, the presence of a tin layer on the surface of the sheet used creates difficulties in the stamping due to the transfer of material from the sheet to the tool, addition of the coefficient of friction, and premature wear of some surfaces of the tool where the contact is more pronounced. In order to understand and avoid these phenomena, a TiAlN coating deposited by a physical vapor deposition (PVD) process was used, which was studied in the laboratory to analyze the evolution of the friction force on the contact and to verify the reaction of the coating on contact with tinplate. Afterwards, the tool was coated and practical tests were performed on the stamping. The obtained results allow confirmation of a significant improvement of the wear behavior of the tool when provided with the coating and also verify that this coating presented better wear resistance than others previously tested in the same working conditions.

## 1. Introduction

The packaging industry represents a very active sector in the market, and packaging can use different materials, such as metal alloys, paper, plastic, and glass, depending on factors as diverse as shape, appearance, and type of storage necessary, among many others [1,2]. It was estimated that 69% of the packaging market concerns food packaging and beverages [3]. The food industry has very specific requirements due to factors such as noncontamination of food, conservation, and cost, as the quantities involved require affordable pricing [4,5]. However, requirements may vary from country to country, or from region to region, depending on factors such as the average weather conditions, legislation, and culture of each people [6]. In fact, food packaging must fulfil four main functions [7,8]: containment, protection and preservation, convenience, and communication. In addition to the functions previously assigned to packaging, there are also growing concerns about the environment, so there are other factors that are now particularly relevant, such as energy, raw materials, recycling, sustainability, and compliance with environmental sustainability [9]. Metal packaging is of particular importance when rigid systems are intended to avoid fracture of food during transportation, storage, and exposure. Within the group of metallic packaging, materials such as tinplate are widely used because they are approved by the organizations that regulate food production [10]. However, tinplate has shown some processing problems during stamping, as tin tends to adhere to the stamping tools, significantly increasing the friction between the main components of the tool (die and punch) and the sheet being molded [11,12]. The transfer of tin or other alloys to the tool or mold can be avoided through the use of coatings made by physical vapor deposition (PVD), which have a low affinity for the uptake of tin by adhesion [13]. Considering tinplate, the surface layer of tin is electrolytically applied in a continuous way. Tin is 100% recyclable, so packaging produced with this material can be considered environmentally friendly, with the added advantage of combining ductility with mechanical resistance, which allows packaging to be easily obtained through the stamping process. The use of this material in packaging is further enhanced by the fact that tinplate has good weldability and high resistance to corrosion [14].

In order to minimize the adhesion of the metals to the stamping tools, some other coatings have been tested in the most diverse situations (NC/NiBN, NC/WC, TiN), analyzing the friction and dissecting the wear mechanisms of the tools associated with this adhesion [15,16,17,18], but the results achieved so far are not sufficient to achieve a long lifespan of the dies in the stamping process as required. Among some coatings that have begun to show clear evidence in resistance to wear, the TiAlN coating is particularly promising. Indeed, the TiAlN coating has been extensively studied for a wide range of applications [19,20,21,22,23,24], but few studies have focused on its use in metal stamping applications [25,26]. The study carried out by Elmkhah et al. [25] concluded that a multilayered TiN/TiAlN/TiN coating showed a better adhesion to the AISI H13 steel substrate than the monolayered TiAlN coating. This effect has been attributed to the hardness gradient provided by the TiN layer between the substrate and the TiAlN intermediate layer. Also, the coefficient of friction exhibited by the TiN/TiAlN/TiN multilayered coating was significantly lower than that presented by the TiAlN coating, being 0.2 and 0.5, respectively. However, the detachment of the external layer of TiN, which leaves the layer of TiAlN exposed and thus allowing the formation of Al_2_O_3_, usually leads to very low coefficients of friction, facilitating slippage. However, if this explanation is valid for this case, it would also be for the case where the entire TiAlN layer is exposed to the environment and may form Al_2_O_3_ even more strongly, a tribo-chemical layer that facilitates the sliding motion, significantly reducing the friction coefficient. In that same work, the authors also measured the hardness of both films, obtaining slightly higher values for the TiN/TiAlN/TiN multilayer coating, which also contradicts the idea previously suggested by the same authors that adhesion was superior in these multilayered films due to the presence of a less hard TiN layer between the TiAlN and the substrate. On the other hand, Cora and Koç [26] also tested TiAlN coatings obtained by a PVD process using a nonreciprocating wear test system in the laboratory, concluding that this coating showed robust wear resistance with very low dispersion of results. The best wear resistance results obtained with the TiAlN coating compared to the TiCN coating tested under the same conditions was attributed to the TiAlN coating having a hardness higher than its competitor in that work. The works previously presented have results essentially based on laboratory results, which do not always conveniently replicate the real conditions obtained during the stamping process.

The objective of this work was to study the feasibility of applying TiAlN coatings in stamping tools devoted to the manufacture of covers for cookie packages. In this stamping operation, transfer of tin from the tinplate to the tool was very common, increasing the frictional forces and generating premature wear of the tool. The service life of the tool between reconditioning operations in the areas most subject to wear was about 80,000 cycles. The coating was initially tested in the laboratory by means considered suitable for this purpose, and after validation of the application viability, was used in the tool and tested in an industrial context.

## 2. Materials and Methods

### 2.1. Specimens Used in Laboratory Wear Tests

**Figure 1:**
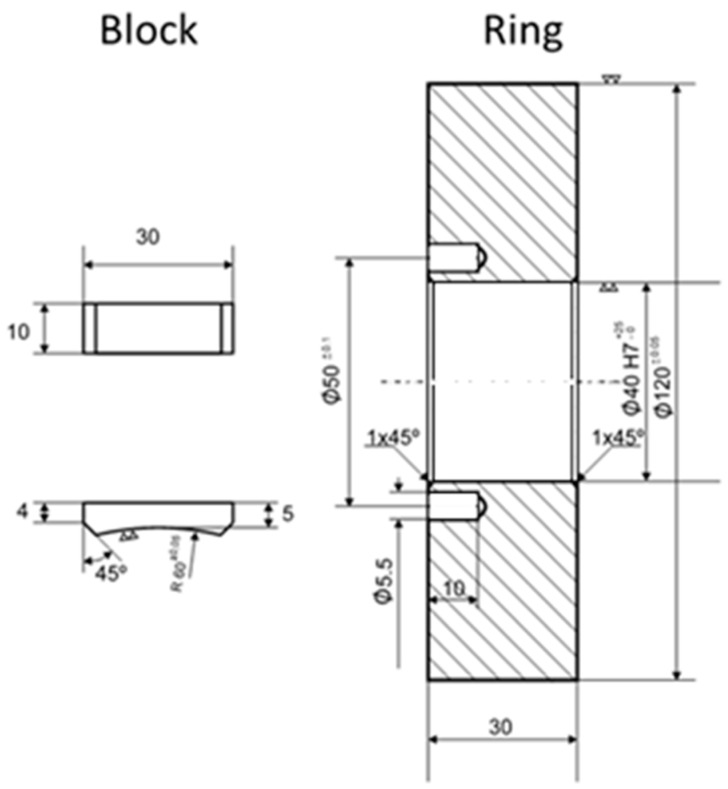
Geometry of blocks and ring used.

In order to firstly obtain results that allowed evaluating the feasibility of applying this type of coating to the stamping tool, a block-on-ring configuration was chosen to carry out tests in a laboratory environment. The configuration of this test can be seen in Figure 1, which is used when it is intended to impose high normal loads on conforming contacts subject to sliding.

The material selected to produce the samples (block and ring) was AISI D2 steel, in order to simulate what could happen in the mold. In order to make the laboratory test closer to the reality that it was intended to evaluate, the surface of the skate was covered with a tinplate plate adjusted to its dimensions, which was glued with a 3M DP8407NS acrylic structural adhesive, suitable for bonding metals and exhibiting sufficient levels of strength and ductility for the loads in question. The arithmetic average roughness of the ring was evaluated, as well as the roughness of the tinplate used over the tinplate surface, and the values obtained were 0.028 µm and 0.138 µm, respectively. The roughness was assessed using a VEECO Multimode atomic force microscope (AFM) in contact mode (7 nm tip radius), evaluating an area of 20 × 20 µm^2^ in each sample. 

Each type of sample was intended to simulate one of the components of the stamping tool: the ring was intended to simulate the surface of the tool, while the block covered by the tinplate was intended to simulate the sheet that would be stamped on the tool mounted on a press.

### 2.2. Film Preparation

The deposition of TiAlN was performed in an industrial CemeCon^®^ CC800/9ML PVD magnetron sputtering machine, achieving an estimated composition of Ti_40_Al_60_N, following the very good results obtained by [13] in a similar context. Substrate surface preparation was carried out first, with samples being cleaned in an ultrasonic degreasing bath for 10 min, followed by a demineralized water bath in order to avoid surface contamination. Detailed parameters of the PVD magnetron sputtering deposition process are shown in Table 1. This reactor allows sample rotation during the deposition process, providing better homogeneity in the film composition. The rotation speed of the substrate holder was 1 rpm. 

### 2.3. Coating Characterization

Coating surface morphology and film thickness were studied using a FEI Quanta 400 FEG scanning electron microscope (SEM) equipped with an EDAX Genesis X-ray spectroscopy system. In order to analyze the TiAlN film thickness, a section was cut, followed by metallographic preparation of the cross section by sanding the samples with a sequence of 320, 600, and 1200 grit sandpapers, followed by polishing operations with 3 mm and 1 mm diamond powder suspension. 

### 2.4. Adhesion Analysis

The adhesion of the films to the steel substrate was evaluated with two different techniques: scratch test and Rockwell indentation. Scratch test was performed using CSM REVETEST scratch test equipment not provided with acoustic emission, according to the BS EN 1071-3:2005 standard [27]. Load range selected was 0–100 N at an increasing rate of 10 N·min^−1^ and an indenter sliding speed of 10 mm·min^−1^, which produces a progressive scratch due to the action of the diamond Rockwell indenter. In order to maximize result accuracy, tests were repeated three times, and the presented results embody the average value. Rockwell test procedure was carried out as specified in the VDI 3198:1991 standard [28]. This test was done by means of an EMCO M4U universal hardness tester operating at 1470 N (150 kgf) with a diamond Rockwell indenter. Indentations were assessed using the same equipment for coating characterization (SEM), and border craters were compared with the illustrations and failure modes shown in the abovementioned standard, allowing the classification of the failure pattern obtained.

### 2.5. Microhardness Test

Hardness characterization was carried out using a Fisherscope H100 microhardness testing machine equipped with a Vickers indenter. The selected normal load was 50 mN with a dwell time of 30 s, in this way avoiding creep phenomenon. This equipment produces ‘load–depth’ curves allowing for the analysis of the hardness (H) and reduced Young’s modulus (Er). This test was performed according to ISO 14577-1:1015 [29] and repeated ten times in different coating surface areas in order to obtain an average value, providing the required accuracy. The collected data were recorded and treated by the resident microhardness software, allowing obtaining the curves and values of the variables under analysis.

### 2.6. Wear Behaviour Characterization

**Figure 2:**
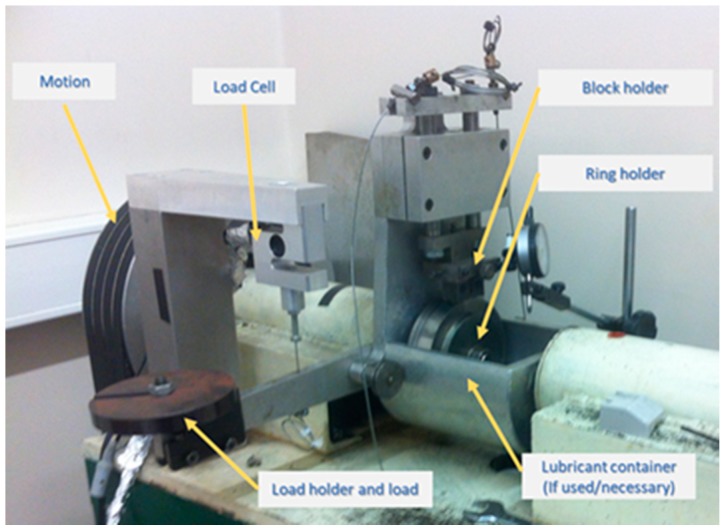
Block-on-ring configuration view.

Wear characterization was assessed using a block-on-ring test configuration (Figure 2). This in-house-made equipment has previously been validated, having been used in several previously published works. The coated ring is intended to simulate the coated surface tool, whereas the tinplate bonded to the block tries to simulate the action of tinplate during the stamping process. The wear test equipment is provided with a load cell which is connected to the equipment arm to determine tangential force induced during the contact between the coated ring surface and the tinplate bonded on the block’s surface throughout the tests. An electronic device filters and magnifies the signal emitted by the load cell, converting it into tangential force, expressed in N. The data is subsequently acquired by a National Instruments (NI-DAQ model PCI-6281, 500 kS/s (multichannel), with programmable 40 kHz lowpass filters) data acquisition board and afterwards is processed and recorded by an in-house-made application based on LabView 4.0 software. The number of cycles and speed were programmed into the controller, allowing reproducibility of results. The number of cycles to be performed by the samples was selected taking into account the high load to which the samples are subjected during the test, following procedures previously used in similar studies [12,13,30]. The summary of the parameters selected for the tribological tests can be found in Table 2.

Experiments were performed three times in order to improve result accuracy, under dry contact conditions using 70 N as a normal load and 0.38 m/s linear speed. The tribological study aims to determine wear behaviour at room temperature, reflecting similar conditions in the industrial process, with the primary purpose of studying possible tin transfer. Knowing that adhesion of tin from steel metal to the stamping tool surface is one of the main wear mechanisms, wear results were analyzed by SEM concerning the material transferred from one surface to the other. Furthermore, wear was evaluated in qualitatively, not quantitively, being the most appropriate type of analysis regarding the main goal of this work.

### 2.7. Stamping Tests

**Figure 3:**
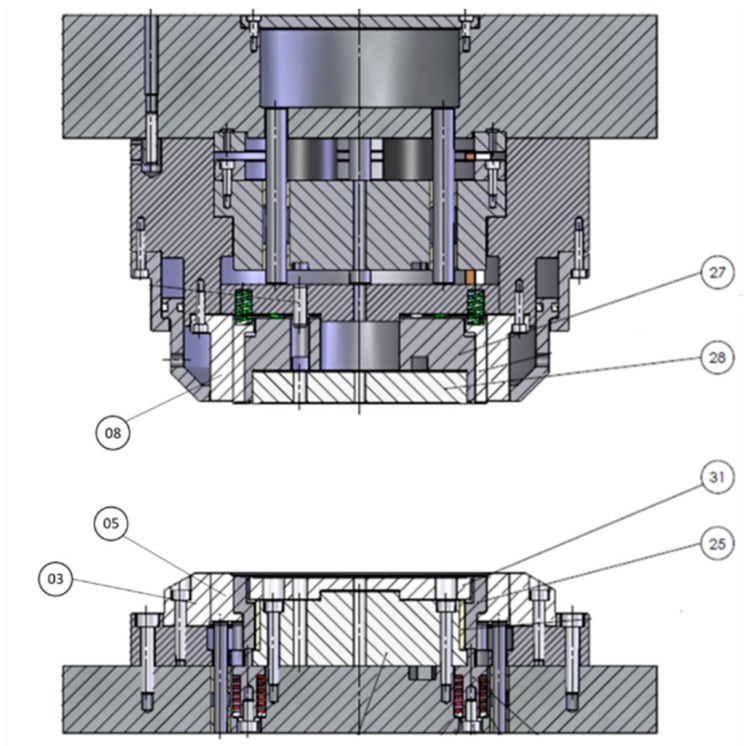
Punch and die drawings, showing their main components: 03—Die; 05—Pressure ring; 08—Cutting punch; 25—Draw ring; 27—Upper draw core; 28—Upper draw insert; 31—Lower draw core insert.

**Figure 4:**
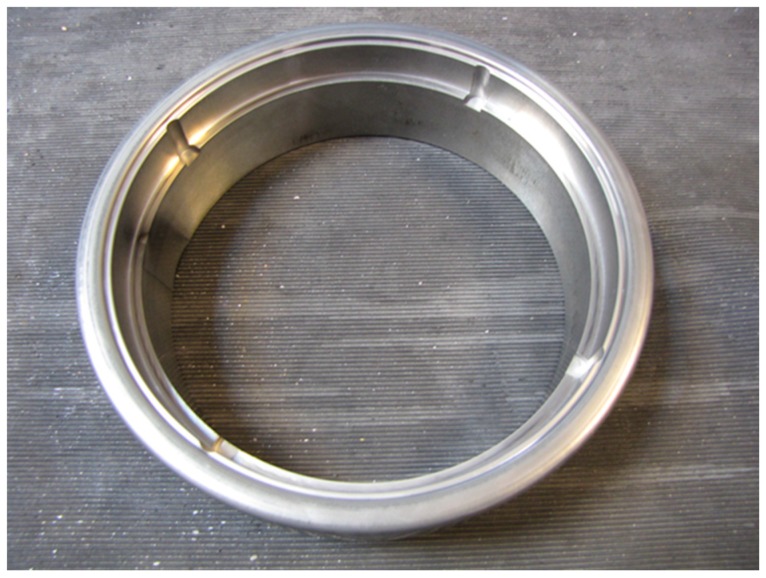
View of the already coated pressure ring which was used in the industrial tests.

In order to perform the industrial wear tests, a 250 kN mechanical press was used with variable speed during the movement of the tool in order to minimize the impact of the upper and lower sides of the tool. In Figure 3, the technical drawing of the tool usually used to produce the covers of the cookies can be seen, with the main components labeled, and in Figure 4, the pressure ring corresponding to component 05 in Figure 3 is shown, which, being the component where the wear was most strongly felt, was coated with TiAlN in the same way as the laboratory samples in order to analyze its wear behavior in service according to the conditions normally used for the stamping of the cover under study.

The pressure ring was made of AISI D2 steel and heat-treated, having a hardness between 52 and 54 HRC, which was verified using the same durometer used to perform the indentations related to the adhesion tests. Surface roughness, measured after polishing and surface coating through a MAHR PETHOMETER M2 probe, allowed the conclusion that the surface arithmetic mean surface roughness was 0.349 µm with a total roughness of 3.618 µm, using a cutoff distance of 0.8 mm.

In order to test the behavior of the pressure ring, 90,000 stamping cycles were performed with the coated tool, and the ring was then extracted to check for wear on the ring. As the friction generated in the tool is converted to heat, a temperature analysis of the pressure ring was carried out by means of thermography, comparing images collected before and after the pressure ring was coated. For this purpose, a Fluke Ti400 infrared camera capable of measuring temperatures in the range of −20 °C to 1200 °C and an associated error of less than 2% was used.

### 2.8. Thermographic Anlysis

In order to monitor the increase of temperature registered during the experimental tests carried out to simulate the industrial environment, a thermographic Fluke TiX560 camera was used, which had the main characteristics of temperature measurement range of −20 °C to 1200 °C and thermal sensitivity of ≤0.03 °C at 30 °C target temperature.

## 3. Results and Discussion

**Figure 5:**
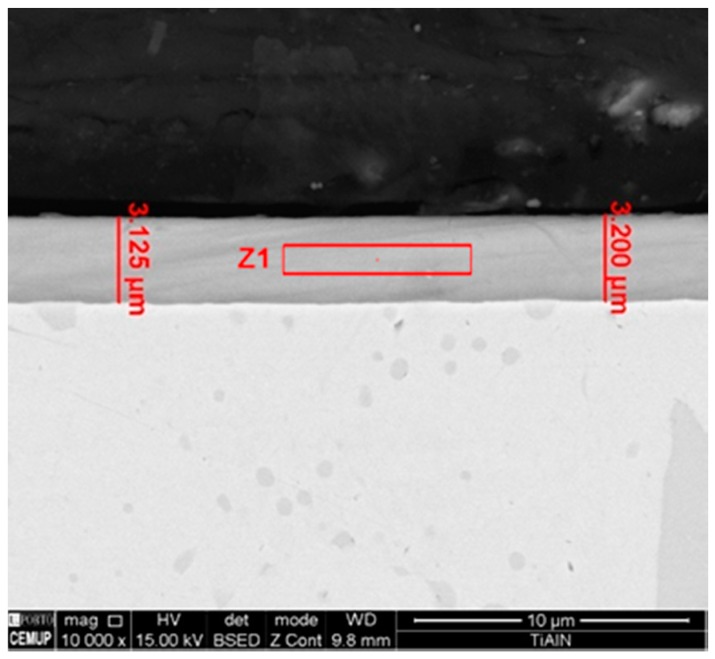
SEM micrograph of TiAlN film cross section with corresponding thickness measurements.

**Figure 6:**
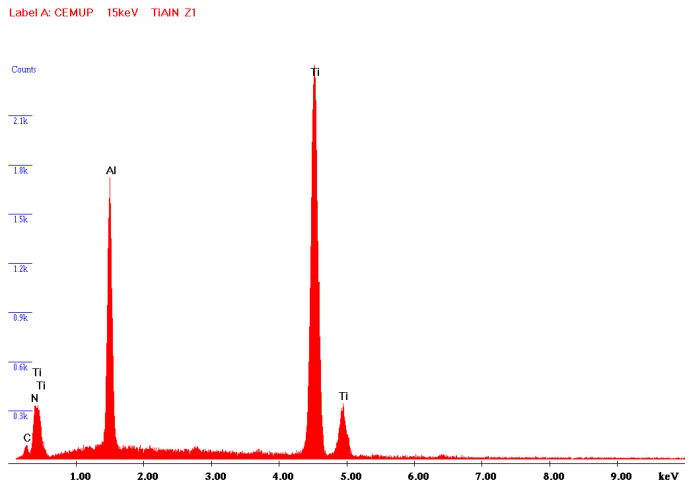
Energy dispersive spectroscopy (EDS) spectrum collected during SEM observations presenting the TiAlN composition.

**Figure 7:**
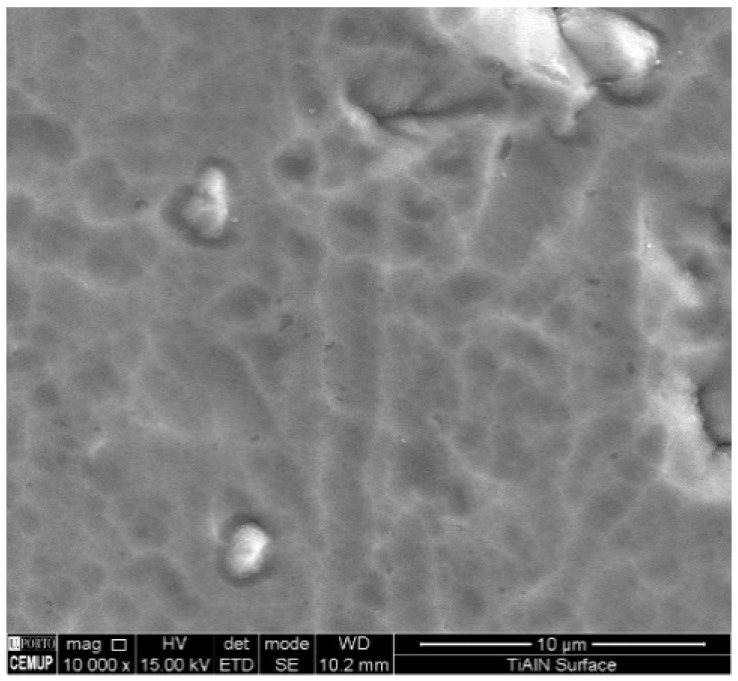
SEM top view of the TiAlN film surface.

**Figure 8:**
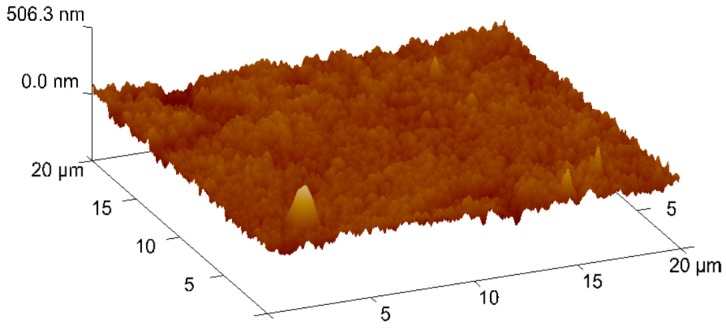
Atomic force microscopy (AFM) 3D analysis of the TiAlN coating morphology.

Information about TiAlN film morphological characterization and thickness of the samples used in laboratory tests was assessed by scanning electron microscopy (SEM), as referred to previously. In Figure 5, it is possible to observe that the film is composed by a thin monolayer with ~3 µm thickness and excellent uniformity. Energy Dispersive Spectroscopy (EDS) spectrum (Figure 6) confirms that TiAlN film composition presents the expected elements and that the film is homogeneous regarding both structure and thickness. Regarding the values provided by the SEM/EDS analysis, excluding the small amounts of other elements such as C, the composition of the film is 58 wt % Ti and 42 wt % Al. This composition contrasts with others presented in different studies [13], showing a composition of approximately Ti_60_Al_40_N. It should also be noted that the intensity of the nitrogen peak observed in this work is lower than that recorded in other studies [13], denoting a lower retention of nitrogen in the structure of the deposited film. Morphology of the film presents a smooth surface, and therefore this morphology is generally more suitable for tribological applications (Figure 7). Surface roughness can be observed in Figure 8, acquired using atomic force microscopy (AFM). The measured arithmetic mean roughness obtained with this technique was 28.4 nm and the maximum roughness was 37.8 nm, using an analyzed area of 20 µm × 20 µm, which would be considered as a very smooth surface.

**Figure 9:**
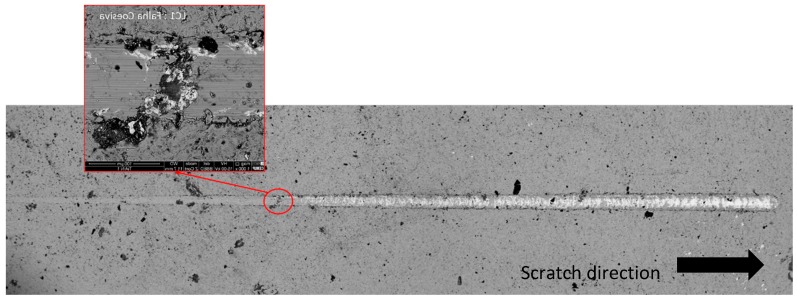
SEM image of one of the three TiAlN coating scratches produced during the scratch tests.

Scratch test was used to assess adhesion between the coatings and substrate. The failure mechanisms were analyzed by SEM for careful observation of the scratch grooves. Each failure mechanism was identified and the corresponding distance to the start point was measured in order to observe the corresponding normal load in the range of 0–100 N. The first cohesive failure was registered at 38 N, showing no detachment at the edge of the scratch, and therefore, no adhesive failure was verified (Figure 9).

**Figure 10:**
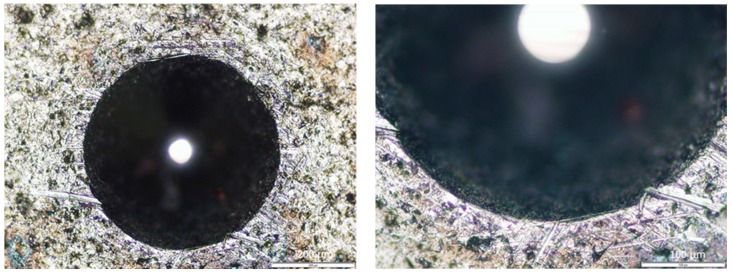
Rockwell indentations carried out at 150 kgf, at 100× (**left**) and 200× (**right**) magnification.

To complement these results, some indentations were also done. Figure 10 presents the result, where it is possible to observe the absence of cracks in the indentation border. This result is consistent with those of the scratch test. According to the VDI 3198:1991 standard [28], these results can be classified as ‘HF1’ behavior. This denotes a satisfactory adhesion pattern, and there is no film detachment at indentation edges. The indentation results can be more accurate than those of the scratch test because they are less influenced by the film hardness, surface roughness, and coating thickness, due to a slower normal load increase. Hence, it is possible to conclude that the coating has good adhesion, an important characteristic for tribological applications.

Due to low film thickness, coating hardness was assessed using an extra low load in the microhardness tester. As is well-known, indentation tests depth cannot exceed 10% of the film thickness, in this way avoiding substrate influence on hardness results [13,31]. Elastic recovery problem was solved by using a dwell time at maximum load of 30 s. Vickers hardness and Young’s modulus of the film was determined by Vickers indentation, and the results are presented in Table 3.

The microhardness obtained revealed that the films analyzed are significantly softer than reported in the literature, which provides values in the range of 21 to 36 GPa [32,33,34]. This difference can be justified by a less compact film structure, different crystalline phases, as well as different structures (amorphous and/or crystalline) or simply the different stoichiometry of the TiAlN coating. 

**Figure 11:**
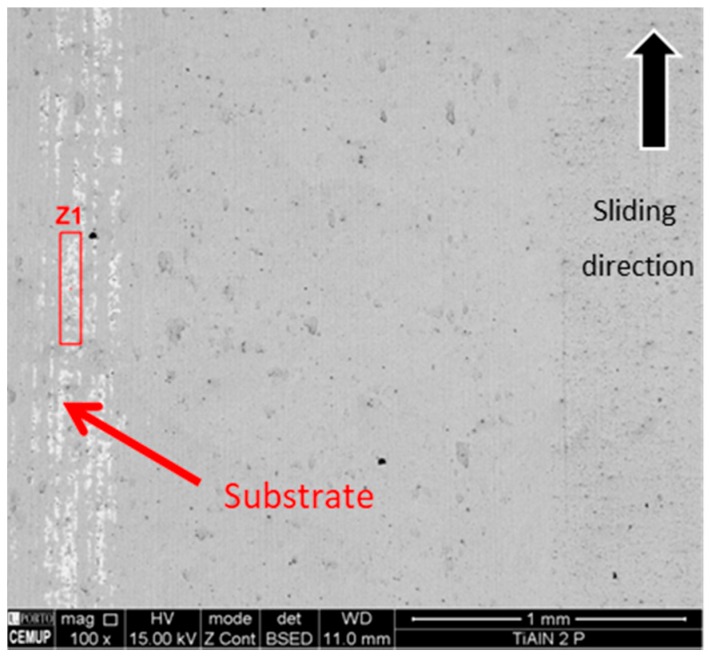
SEM image collected on the coated ring surface after tinplate contact during the wear tests.

**Figure 12:**
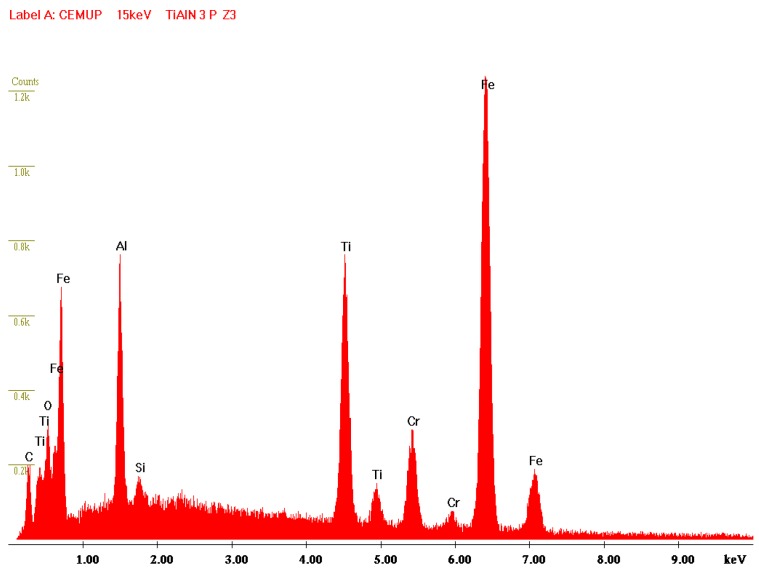
Spectrum of the TiAlN surface at area Z1.

A material transfer between samples was expected due to the hardness difference between surfaces, increasing the weight on the harder surface due to the transfer of the softer material from the tinplate. For that reason, a quantitative evaluation of mass loss was not measured. Furthermore, tin transfer from the tinplate surface to the coated ring was considered more important than weight loss. SEM images of scars and grooves collected from the coated surface (ring) are presented in Figure 11. It is possible to observe some scratches following the direction of motion. Furthermore, it is possible to observe the absence of formation of adherent films on the coating surface, although TiAlN presents a slight film wear exposing the substrate, as can be confirmed by the EDS spectrum depicted in Figure 12. In the SEM image (Figure 11), it is also possible to observe a central area where slight wear traces can be identified, and on the left side area, a much more severe wear is identified, so critical that it exposes the substrate. Considering the outcomes obtained, it is possible to state that TiAlN seems to be suitable for tribological applications, although an increased wear resistance would be necessary regarding the laboratory tests carried out. Due to a larger content of aluminum in the TiAlN coating, the hardness of the coating presents lower values than the usual hardness pointed out by similar research works based on the same coating. Thus, it is expected that by adjusting the coating composition, the wear results can be improved.

**Figure 13:**
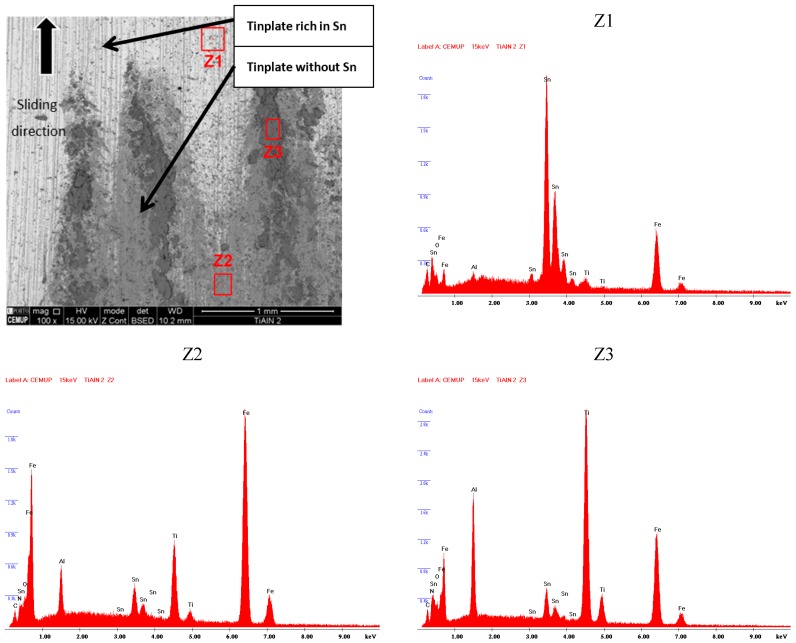
SEM observation and spectra of transferred TiAlN coating particles.

Each tinplate sample bonded to the block was removed for observation in SEM as well. During the tests, transference of detached coating particles from the coated ring surface to the tinplate surface had occurred. The transferred material was identified and characterized using EDS, aiming to detect the type of elements present on the tinplate surface. Being a conformal-type contact, there is an area where the contact seems to be more intense, and there is some movement of soft tin being kept on the peripheral area, as can be observed in Figure 13. As observed previously, the TiAlN coating has good adhesion to substrates combined with an unexpected low hardness, and for these reasons, during the contact, oxide sludge is generated, meaning the perfect distinction of TiAlN particles is not possible. Regarding Figure 13, it is possible to state that area Z1 is predominantly constituted of tin, showing that the tin film remains in the tinplate surface. In the Z2 area, the presence of tin film is very reduced, with the peaks of iron and the presence of particles from the TiAlN coating being noticeable, as can be proved by the peaks of Ti and Al, which can be observed in the Z2 EDS spectrum. Tinplate is material transferred from the TiAlN coating, confirming as well that this film is altered; area Z3 shows strong peaks of Ti and Al, indicating clearly that the dark surface layer over the film is softer than has been reported by other authors, as mentioned above. 

**Figure 14:**
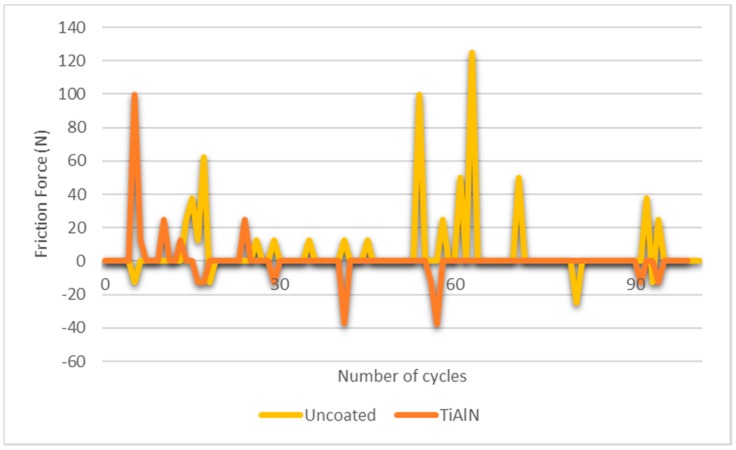
Tangential force evolution over time in block-on-ring tests.

A graph was drawn (Figure 14) regarding tangential load values achieved. With regard to the TiAlN coating, it is possible to observe that with up to 30 cycles, there is a large variation of the tangential force, showing that an initial period of adaptation of the surfaces is required. After that, the friction force becomes much more stable, revealing that the main surface asperities were already removed from the coating surface, an initial tin transfer was already promoted, and due to the heat generated by friction, the oxygen of the air already stimulated the oxides formation, leading to steady behavior until the end of the test.

Regarding the friction of coefficient (COF) analysis, the results obtained with this study indicate a COF of 0.18, slightly lower than that reported in the literature (0.25). Indeed, Chauhan et al. [35] gathered information on studies about mechanical properties taking into account TiAlN-based coatings. Also, Bukhaiti et al. [36] developed studies on the tribological properties of a TiAlN multilayer coating. Thus, the TiAlN coating seems to exhibit a stable behavior over time, with low tangential force variation as well as an outstanding friction coefficient value, which leads to the conclusion that this coating is suitable for industrial stamping processes of tinplate.

After the laboratory characterization of the TiAlN coating, and given the expectations created around its application in the stamping process due to excellent laboratory results, the pressure ring of the stamping tool was coated using the same deposition conditions used for the samples previously tested in the laboratory.

**Figure 15:**
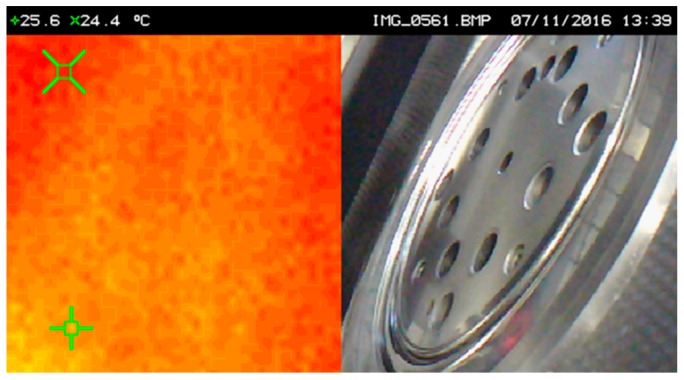
Temperature developed in the uncoated pressure ring.

**Figure 16:**
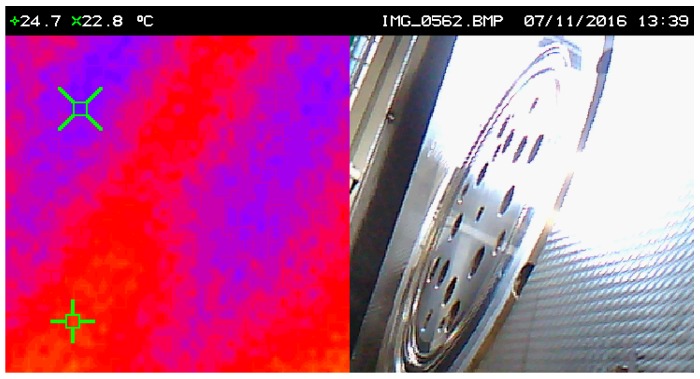
Temperature developed in the TiAlN-coated pressure ring obtained by thermography. In the (**left**) image, the thermograph image corresponding to the tool surface shown in the picture (**right**).

In order to compare the heat generated by friction between the uncoated and the coated tool, a temperature measurement was carried out using an infrared camera in the same working conditions for both situations. Figure 15 and Figure 16 show the images relative to the temperature analyses performed. As verified in the images, after 18 min of continuous work in stable and equal ambient conditions for both tests, the uncoated pressure ring develops a temperature of 24.4 °C, while the pressure ring coated with TiAlN only develops that of 22.8 °C, i.e., about 2 °C lower, thus revealing that the TiAlN coating promotes a reduction of the frictional force developed between the tool and the tinplate, which is reflected in the temperature difference referred to above. It is thus proven in practice, through experimental tests in an industrial environment, that the TiAlN coating favors the contact between the thermally treated steel of the tool and the tinplate. 

**Figure 17:**
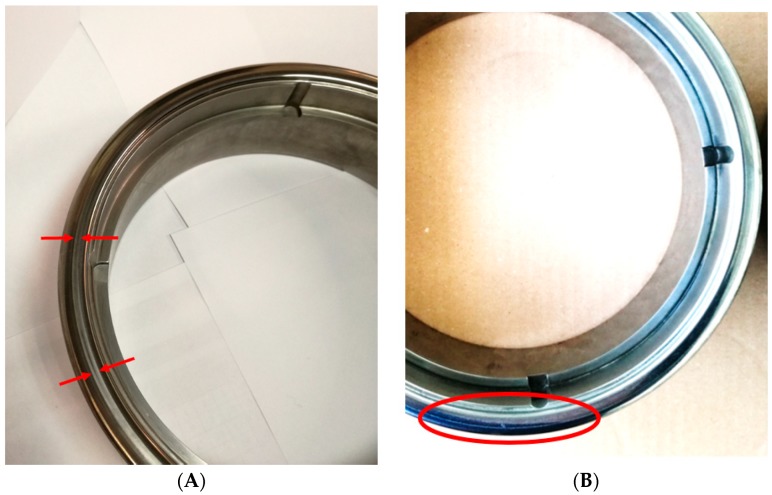
(**A**) Minor wear signs (indicated by red arrows) on the TiAlN-coated pressure ring after 90,000 stamping cycles; (**B**) Uncoated pressure ring after 90,000 stamping cycles with clear tin transfer on the surface and wear marks (indicated by red oval).

The TiAlN-coated pressure ring was also extracted from the tool after 90,000 stamping cycles to identify possible signs of wear. Figure 17a shows the pressure ring with almost imperceptible marks of wear. Given these wear marks and the number of cycles already performed, it is expected that the tool will be able to perform 500,000 stamping cycles until reconditioning is needed and replacement of the coating layer is required. This value was expected based on the low friction coefficient registered and decrease of temperature observed on the surface tool, taking into attention the values mentioned above and industrial experts’ experience.

Regarding Figure 17b, where the uncoated pressure ring presents a severe accumulation of tin at its contact surface, it is usual that this phenomenon leads to severe wear in a short period of time, leading to a detachment of the tool surface.

## 4. Conclusions

This work aimed to evaluate the suitability of TiAlN coating to be used in low-deep stamping tools devoted to working with tinplate. Laboratory tests were considered appropriate to analyze the feasibility of the application of TiAlN coatings in stamping tools, which showed that this type of coating had appropriate characteristics for this type of application. Indeed, the properties of the coating and the way it behaved in the wear tests, exhibiting a low coefficient of friction, have led to the conclusion that this coating has great potential to be applied in soft material stamping tools.

Thus, the work proceeded to the second phase, in which a pressure ring of the tool was coated with TiAlN. This pressure ring had previously shown strong signs of wear and transfer of tin from the tinplate to the pressure ring surface, and through this work, it was possible to confirm that the TiAlN coating significantly improves the life of the tool. Indeed, the uncoated pressure ring required reconditioning every 80,000 cycles, and now, based on the wear reported on the component in analysis after 90,000 cycles, it is expected that the pressure ring needs to be reconditioned only after about 500,000 cycles, which represents a lifetime gain of more than 500%. In addition to the estimated saving in maintenance operations, the coating will reduce tool unavailability and will help the tool life cycle become much higher, avoiding premature resource consumption (even through recycling) while using environmentally friendly coatings.

It has also been experimentally proven that the friction developed by this coating is much lower (COF_coating_ = 0.18) than that obtained in the contact between the steel and the tinplate (COF_steel/tinplate_ = 0.74), which favors the process. Thus, relative to other coatings previously tested for the same purpose, the TiAlN coating presents truly promising results for the intended purpose. However, the composition and stoichiometry of the coating shall be taken into account in order to maintain the hardness at controlled levels, while also maintaining the friction levels below those usually reported for other TiAlN coatings.

## Figures and Tables

**Table 1:** Deposition parameters.

Parameter	TiAlN
Time deposition (min)	180
Chamber gases (min)	Kr—120 mln; Ar—180 mln; N_2_—120 mln
Temperature (°C)	480
Pressure (mPa)	600
Current density (A)	10
Bias (V)	−90

**Table 2:** Tribological parameters used in block-on-ring configuration.

Description	Parameters
Normal load (N)	70
Ring rotational speed (rpm)	60
Ring linear speed (m/s)	0.38
Ring diameter (mm)	120
Contact area between block and ring (mm^2^)	255
Contact persure (MPa)	0.27
Number of cycles	100
Total distance per test (m)	~37.7

**Table 3:** Ultra-microhardness and Young’s modulus of the TiAlN film.

Coating	Hardness	Young’s Modulus	
	H (GPa)	Er (GPa)	E’ (GPa)
**TiAlN**	13.2 ± 3	236.2 ± 34	287.2 ± 55
